# Transactions of the Central States Dental Association

**Published:** 1865

**Authors:** W. H. Shadoan

**Affiliations:** Louisville, Ky.


### TRANSACTIONS OF THE CENTRAL STATES DENTAL ASSOCIATION.

*Louisville, Ky., April 18th,* 1865.

A large number of practicing Dentists met pursuant to a call, at the National Hotel, in the city of Louisville, Ky., on Tuesday, April 18, 1865, at 2 o'clock, P. M.

On motion:

Dr. W. H. Morgan was elected temporary chairman, and Dr. W. M. Rogers, Secretary.

Remarks of Dr. Stone:

GENTLEMEN: Some time ago, several eminent dental practitioners met and held a consultation as to the propriety of organizing a Dental Association, to be composed of all dental practitioners in good standing in the States most contiguous, and to ascertain the opinions of the leading dentists. A memorial was drawn up and signed.

The memorial reads as follows:

The undersigned, practitioners of dentistry, believing that an association of dental surgeons composed of the Central States of North America, would be calculated to promote the best interests of the profession, respectfully suggest that all dentists of the Central States (comprising the States of Kentucky, Indiana, Ohio, Tennessee, Illinois and Missouri,) and all others who are friendly to the advancement of dental science; who can, will meet in the city of Louisville, on Tuesday, April 18th, 1865, for the purpose of forming, if those present deem it advisable, a dental association, for the advancement of the profession in this locality.

N. CLUTE,
W. G. REDMAN,
W. D. STONE,
W. H. SHADOAN,
J. A. MCCLELLAND,
E. W. MASON,
S. GRIFFITH.

On motion of Dr. Shadoan:

*Resolved,* That a committee of three be appointed to draft a constitution and by-laws.

The chair appointed upon that committee Drs. Redman, Shadoan and Rogers.

On motion of Dr. Redman:

Dr. Morgan was made chairman of the committee.

An invitation was extended by the Louisville Dental Association for all visitors to meet with them at their regular meeting, at 7½ o'clock, P. M., at the office of Dr. Stone.

On motion, adjourned to meet at 10 o'clock, A. M., the day following.

SECOND DAY, 10 O'CLOCK, A. M.

The asssociation met according to adjournment.

Minutes of the previous meeting were read and approved.

The committee on constitution made their report as to the constitution, which was adopted by section, and signed by the following members:

(As the constitution is very much the same as those governing dental societies, I shall leave it out of this report.)

*Secretary.*

Drs. W. H. Morgan, W. M. Rogers, S. Griffith, W. D. Stone, W. H. Shadoan, J. A. McClelland, W. G. Redman, G. B. Fritz, L. E. Wilson, C. E. Dunn, J. H. Bedford, John Ragland, S. Driggs, G. S. Jones, J. W. Baxter, R. H. Wilson, B. M. Gildea, S. J. Cobb and J. F. Canine.

On motion:

The special business of the afternoon session shall be the election of officers.

On motion:

Adjourned till 2½ o'clock, P. M.

2½ O'CLOCK, P. M.

Minutes of the previous meeting were read and approved.

On motion:

The society went into the election of officers for the ensuing term, which resulted as follows:

*President*, Dr. W. H. Morgan, of Nashville, Tenn.

1*st Vice-President*, Dr. S. Driggs. of Louisville, Ky.

2*d Vice-President*, Dr. W. G. Redman, of Lexington, Ky.

3*d Vice-President*, Dr. J. W. Baxter, of Warsaw, Ky.

*Secretary*, Dr. W. H. Shadoan, of Louisville, Ky.

*Cor. Secretary*, Dr. J. A. McClelland, of Louisville, Ky.

*Treasurer*, Dr. R. H. Wilson, of Louisville, Ky.

The following were appointed the standing committees:

*Executive Committee.*—Drs. Stone, Cobb and Griffith.

*On Membership.*—Drs. Driggs, Rogers and Baxter.

*Publication Committee.* Drs. Shadoan, Fitz and Dunn.

ESSAYISTS.

*Operative Dentistry.*—Dr. J. A. McClelland.

*Mechanical Dentistry.*—Dr. R. H. Wilson.

*Dental Ethics.*—Dr. B. M. Gildea.

*Dental Medicine.*—Dr. W. G. Redman.

*On Dental Education*, Dr. J. F. Canine.

On motion:

Drs. Redman and Gildea were appointed a committee to conduct the officers elect to their places, which was done in a very becoming manner.

On motion:

Adjourned to meet at the office of Dr. Shadoan, at 7½ P. M.

7½ O'CLOCK, P. M.

Society met according to adjournment.

Minutes read and approved.

The committee on by-laws made a report which was adopted, and the committee held over till the next meeting to finish their report. (The by-laws are omitted in this report for the same reason as the constitution.)

On motion:

Adjourned to meet at the office of Dr. Redman, at 9 o'clock, A. M.

THIRD DAY.—9 O'CLOCK, A. M.

Association met according to adjournment.

Minutes of the previous meeting were read and adopted.

Several communications were read from members of the profession, apologizing for their non attendance, and promising to be present at our next meeting.

On motion of Dr. Stone:

Their apology was accepted, and an invitation extended them for the next meeting.

On motion:

Professor Frazer was elected an honorary member of the Central States Dental Association.

On motion of Dr. Stone:

Dr. Shadoan was appointed a committee to prepare and send to the *Dental Register of the West* and the *Dental Cosmos* the proceedings of this society, with a request that they publish them.

On motion:

The afternoon meeting will be to witness a clinic from Dr. Fritz at his office.

Adjourned to meet at the office of Drs. Wilson and Fritz, at 2½ o'clock P. M.

2½ O'CLOCK, P. M.

Society met at the office of Drs. Wilson and Fritz.

All took deep interest in the filling of teeth by Dr. Fritz with the mallet.

7½ O'CLOCK, P. M.

Society met at the office of Drs. McClelland and Canine.

Minutes were read and approved.

The organization being fully perfected, and there being so little time to discuss any particular subject,

On motion:

Each member was requested to make a brief report of some interesting case that had come under his own observation, which resulted in some very interesting reports.

I would take pleasure in sending these reports, if those making them would favor me with a statement of them, but as I did not make a note of them at the time, I do not recollect enough of them to report them.

The hour of adjournment having arrived, a motion was made and carried, tendering the thanks of all visitors to the Louisville Dental Association for their kind hospitalities tendered them in the shape of an elegant entertainment at the St. Charles.

On motion:

Adjourned to meet in this city on the third Tuesday in July next, at 10 o'clock, A. M.

W. H. SHADOAN, *Secretary.*

